# The Medical Society of the County of Erie

**Published:** 1905-03

**Authors:** Franklin C. Gram


### SOCIETY PROCEEDINGS.

### The Medical Society of the County of Erie.

### EIGHTY-FOURTH ANNUAL MEETING.

Reported by FRANKLIN C. GRAM, M. D., Secretary.

THE Medical Society of the County of Erie met in the rooms of the Society of Natural Sciences, Buffalo Library Building, Tuesday, January 10, 1905, at 10.30 a. m., for its eighty-fourth annual meeting, the president, Dr. William C. Krauss, in the chair.

The minutes of the previous meeting were read by the secretary and approved.

Dr. William Warren Potter, chairman of the Membership Committee, reported favorably on the application of Dr. James Francis Rice, who was thereupon elected a member.

Dr. Edward Clark, treasurer of the society, reported a balance of $30.58 in the treasury, submitting also a statement of receipts and expenditures during the year. The report was referred to an auditing committee, composed of Drs. J. H. Grant, F. E. Fronczak and P. H. Hourigan, who approved the same.

The president appointed as a Nominating Committee, Drs. Henry Lapp, William Warren Potter, C. W. Bourne, C. E. Ernest and James Stoddart. This committee presented the following nominations: president, John D. McPherson; vice-president, A. H. Briggs; secretary, Franklin C. Gram; treasurer, DeWitt C. Greene; censors, Henry R. Hopkins, chairman, J. H. Grant, Irving W. Potter, Ernest Wende and F. E. Fronczak. The candidates were unanimously elected.

Dr. William Warren Potter stated that Dr. Edward Clark, who had held the office of treasurer for ten years, had declined a reëlection, and on his motion a vote of thanks was tendered Dr. Clark for valuable services faithfully performed.

Dr. Henry R. Hopkins, chairman of the Board of Censors, submitted the following reports:

*To the President of the Medical Society of the County of Erie:*

Mr. President—Your censors would respectfully report that during the year 1904 the work of the censors has been chiefly directed towards the effort of limiting, discouraging, making unpopular and unprofitable, the criminal practice of medicine in the county of Erie.

Questions of ethics and instances of ethical violations have been brought to our notice, but have not engaged our extended attention for the reason that it seemed to us that our paramount duty was the discouragement of the criminal practitioners of medicine as before stated. Perhaps a word of explanation is here in order, that there may be no misunderstanding as to the significance of the term criminal practice of medicine as the same is used in this report.

The criminal practice of medicine is any and all of the practice of medicine contrary to the provisions of the public health law of the state of New York, the same being known as chapter 25 of General Laws. The persons whose crimes are defined by section 153 of this law are of two classes, misdemeanants and felons, and their several crimes are accurately defined and their several penalties noted.

It will be observed that this is a distinct inclusion within the criminal class of many others than those who are wont to commit such well-known criminal acts as fatal poisoning, maiming, blinding or criminal abortions, and makes the unauthorised, the unlicensed practice of medicine a crime, or even the attempt to practise medicine by appending the letters M. D. to the name, by assuming the title of doctor in such a manner as to convey the impression that one is a legal physician also a crime.

Again, the laity and many of our profession hold that the contentions of doctors even the prosecutions of the censors originate hair-splitting distinctions as to theories concerning modes of medicine giving, or theories of still less practical value as to principles of ethics and etiquette between doctors, or doctors and laity; and so holding, have scant patience with the same, and almost universal sympathy for the party attacked.

It has been the purpose of the censors during the year, and is the purpose of this report to set forth so plainly that all may see and may understand that this society is not before the public, or is not asking the attention of the prosecuting officers of the county, or the time or attention of the courts, with questions of manners, of etiquette, of ethics, but, on the contrary, is solely engaged in the arrest of medical criminals, the prevention of the criminal practice of medicine.

Frankness compels us to state that our success has not been equal to our hopes, although not far from our expectations, we have been successful in many cases, but the criminals who have been brought to punishment are few and insignificant in comparison with the number who are prospering and waxing fat in the successful prosecution of their evil ways.

The public health law, the wisest and the most humane of our statutes, has been and is openly, flagrantly and defiantly violated day by day by scores of criminals, notwithstanding the best efforts of this society continuously exerted for the ten years last past.

What is true of the medical criminals of Erie county is also true of the medical criminals of the other counties of the state in proportion to the number and means of the population.

The incentive which prompts the medical criminal to a life of crime is the usual incentive peculiarly operative upon the criminal class at large,—the money there is in it. We have heard in well nigh every case, the same excuse, palliation and defence, "I would not do this, but I need the money." How powerful this incentive to crime may be was shown in the case of Antonius the Boy Wonder, now serving sentence in the Erie County Penitentiary. At the time of his conviction it was estimated by those having opportunity to know, that his criminal work was yielding him an income of more than $50,000 a year.

In the interest of accuracy often akin to morality, it should be stated that Antonius's income had to be shared with a large number of associates and confederates.

Criminal practices of the Antonius class are only possible by the most persistent and continuous advertising chiefly through the columns of the daily press, and we are informed that the style of advertising suitable for their purpose is the most expensive to be had, and we may safely conclude that the gross income of an Antonius is quite different from that which remains after his associates and confederates in crime have had their several shares of the plunder.

However, at this point it should be noted that the psychology of the degenerate, the criminal class, operates powerfully to the promotion of crime. The criminals, the degenerates, the insane are known to have brains and minds of structures and functions with much in common. They are made alike, they think, feel, and act alike, and an exalted, pathological egoism is common to them all.

It is quite probable that the average criminal would feel that his money was well spent so long as by spending it he could see his picture in the columns of the daily paper and could read of his self-asserted enormous abilities, his marvelous powers, his miraculous works, his God-like personality. When to this is added the certainty that such publications will bring to him victims to be fleeced, the credulous to be imposed upon, the easy who will give up their money, we think we have made it clear why the medical criminal abounds and why his crimes are both notorious and persistent.

We should hate, detest and abhor crime; in this direction, it may aid some of us to remember that the victims of the medical criminal are almost invariably the poor, the ignorant, the afflicted, the most helpless of all our dependent people. The burglar and the highwayman are liable at any time to encounter an able-bodied and possibly an armed man. These criminals must at least have the courage of their depravity. The medical criminal of the popular, the advertising, the Antonius type, robs the poor, the sick, the deformed, the deaf, the blind, the helpless.

If public opinion were quite moral, healthful and sane, the medical criminal would soon find his vocation dangerous and unprofitable, his associates and confederates would be ashamed and forsake his companionship.

To produce this improved state of public opinion, to enforce the humane provisions of the public health law, are the more important purposes and objects for which state and county medical societies are made and provided.

In conclusion, your censors commend this portion of your task, the execution of the medical law of the state as the same applies to the county of Erie, as an opportunity to do effective work in the promotion of the public health of great potentiality, of enormous responsibility.

With keen regret we invite your attention to the general unpreparedness of the medical profession for the proper discharge of this important trust. The medical criminals, misdemeanants and felons have much in common with the grafters and seldom war upon each other. The medical profession should stand with a solid front between the medical criminals and the public; and the medical profession is in four hostile parties, one of 1806; one of 1857; one of 1865, and one of 1900, so designating them with reference to their respective state charters. These four divisions of our profession, or schools of medicine socalled, are fighting for rival opinions as to the dose or source of remedies, as to questions of etiquette or ethics,—and in the meantime the medical criminals are flourishing mightily.

The detection and punishment of crime means the employment of legal counsel and of detectives; this means the expenditure of money. During the year of 1904 your board of censors has received from your treasury, not one dollar. We have raised by subscriptions from wise and law-loving members of the laity, for our work, the sum of $185.00.

It is but simple justice that we make public acknowledgment of the fact that our counsel John Lord O'Brian, Esq., is much overworked and underpaid. He has given to your work at all times prompt attention, quick sympathy and wise advice.

His most sufficient reward has been the consciousness of duty done by a free citizen to a free state. We trust that in time he will have more material reasons for feeling that this society rightly appreciates his services.

Recognition should also be made of valuable services freely given by the Buffalo police through their efficient and courteous chief, General W. S. Bull. Without the assistance of the police in obtaining evidence, some of our work would have been impossible.

Finally, we desire earnestly to commend and sincerely to congratulate and thank our public-spirited citizens and incorruptible and fearless prosecutors, District Attorney Coatsworth and Assistant District Attorney Abbott. Their sympathy and encouragement have been invaluable, their successes phenomenal and inspiring. The medical profession and the people owe them an everlasting debt of gratitude.

Respectfully submitted,

H. R. Hopkins, *Chairman,*
Henry Lapp,
John H. Grant,
Marshall Clinton.

January 10, 1905.

Favorable comments were made by members on these reports, which, on motion of Dr. Callanan, were received and ordered printed, copies likewise to be given the daily press.

On motion of Dr. William Warren Potter the following resolution was adopted:

*Resolved,* That the thanks of the Medical Society of the County of Erie be and are hereby tendered to the Board of Censors for effective and indefatigable services to the profession and public, and that the thanks of the society be also extended to John Lord O'Brian, counsel, for faithful and meritorious services.

Dr. EDWARD CLARK presented the following resolutions which were adopted:

WHEREAS, The president of the New York State Optical Society has announced that the bill to define and regulate the practice of optometry, which failed to pass at the last session of the legislature, will be brought up again for consideration; and,

WHEREAS, The claim is falsely made that 75 per cent. of the general medical practitioners favor this measure; and,

WHEREAS, We believe from the publications of the opticians who favor this bill that they not only desire to, but are now actually engaged in the practice of a branch of medicine in violation of the present medical law; therefore, be it

*Resolved,* That the Medical Society of the County of Erie, in meeting assembled, protests against the enactment of such a bill as contrary to the best interests of the people of the state of New York.

*Resolved,* That a copy of this protest be forwarded to each senator and assemblyman representing this county.

*Resolved,* That the committee on legislation take such action as may seem best fitted to accomplish the defeat of this and similar measures.

President KRAUSS called Dr. T. M. Johnson to the chair and delivered his presidential address on "The Need of a Medical Library in Buffalo." (See page 499).

On motion of Dr. H. R. HOPKINS a vote of thanks was tendered the retiring president, and on motion of Dr. A. A. Hubbell, the president was directed to appoint a committee of five, with Dr. Krauss as chairman, to investigate the feasibility of a medical library for Buffalo, and to report at the June meeting.

Memorials for members, who died during the past year, were then presented. At the request of Dr. W. C. Phelps the beautiful tribute by Dr. A. A. Hubbell, on the late Dr. Henry D. Ingraham, published in the July number of the BUFFALO MEDICAL JOURNAL, was placed on record by the society.

Dr. Henry Lapp read the following memorial of

REUBEN S. MYERS, M. D.

The hand of death has again entered our ranks and removed one of our most respected and beloved citizens. Only six months ago and he who is the occasion of our sorrow met with us in this room and took an active interest in the deliberations of this society. On September 30, 1904, died Dr. Reuben S. Myers, a well known and respected citizen of Clarence Center, N. Y., at the age of 65 years. Dr. Myers was a son of Jacob and Anna Myers and was born on the 24th day of February, 1839, at Montville, Pa. He was educated in the common schools and graduated from the Millersville Normal School of Lancaster County, Pa. He commenced the study of medicine in 1856 in the office of Dr. Amos K. Rohrer, an eminent physician and surgeon of Montville, Pa. He attended lectures at the medical department of the University of Pennsylvania during the sessions of 1858 and 1859 and later received his degree of doctor in medicine from the University of Vermont.

Dr. Myers was in the active practice of medicine at Clarence Center from 1860 until a short time before his death. He was a member of the Medical Society of the State of New York, the New York Pharmaceutical Association, the Medical Society of the County of Erie, and of the Gross Medical Club, composed of some forty physicians in Erie County. He was always a regular attendant at all meetings of the societies of which he was a member and his genial manner and commanding presence was always welcomed by all members. He was an efficient secretary of the Gross Medical Club continuously for about ten years.

Dr. Myers was the soul of honor in consultation with his medical associates and was never known to do an unprofessional act to a professional brother. In his long country practice he was often thrown on his own resources and was obliged to perform many surgical operations such as removal of tumors, amputations, ligation of important arteries. He made a specialty of surgical work and the treatment of lung and throat diseases.

The doctor was also the author of several valuable papers, one on Albuminuria and one on Puerperal convulsions, published in the *Philadelphia Medical Record.* He was also the author of the following papers, published in the *Olive Tree:* Leprosy, 1887; Drainage, 1888; Cholera, 1888.

In politics the doctor was always an earnest democrat, ever working for the success of his party, and his party in turn always honored and respected him. He was an intimate friend of ex-President Cleveland and in 1870 he was elected coroner, being on the same ticket with the ex-President, who was elected as sheriff of Erie county. During both administrations of Cleveland, he was appointed postmaster at Clarence Center. In 1893 he was appointed by ex-Governor Flower as one of the commissioners for loaning certain moneys of the United States in the county of Erie, which office he held until 1903. He was married to Marion C. Vantine, of Clarence, August 28, 1862. The union was a very happy one and there were born to them six children, three of whom died in infancy. His widow and two sons, Henry S. and John B. and one daughter, Mrs. Kate M. Schaad, all of Clarence Center, survive him. He was a true and affectionate husband, a loving and self-sacrificing father, and it was in his home-life that his true manhood was shown best, always working to make home happy by doing little acts of kindness to those dependent on him. Not only did he endear himself by his kindly manner to his immediate family, but to all with whom he came in contact as a loving and true physician. From all sections there has been extended to his bereaved family the assurance of their appreciation of his services, both as a physician and as a citizen, and their great sympathy for them in the irreparable loss they have sustained. He was a member of Akron Lodge F. and A. M., and in 1862 was the first initiatory member accepted into the lodge after its organisation.

The funeral services were held from his late home in Clarence Center on Monday, October 3, and were very largely attended. The services were in charge of Akron Lodge. The Akron Masonic Quartette rendered appropriate music. Dr. A. P. Meeker, of Clarence, preached the sermon. The pall bearers were Drs. Henry Lapp, J. D. Macpherson, A. F. Erb, C. A. Tyler, Joseph Lehman and W. H. Baker, all members of the Gross Medical Club. And thus was laid to rest a body completely worn out by long and continuous service in the medical profession. All who had the pleasure of knowing Dr. Myers in his lifetime will join in saying, "He did what he could."

Dr. Grover Wende presented the following tribute to

### Samuel H. Lynde, M. D.

Dr. Samuel H. Lynde died at his home in the city of Buffalo, Tuesday morning, October 25, in the thirty-sixth year of his age. Early in his professional life he suffered from a severe and prolonged attack of influenza, from the effects of which he never fully recovered, but through whose gradual complications life became a burden. It did not seem so strange, therefore, that after an illness of one week he finally succumbed to pneumonia, a malady which his condition invited.

Dr. Lynde was born in Buffalo, June 13, 1869. He was the son of Burdett A. and Georgia D. Lynde and received his academic education in his native city. He studied medicine at the University of Buffalo from which he was graduated in 1889; subsequently served as interne at the Buffalo General Hospital; directly afterward went to Wanwantosa, Ill., where he engaged in hospital work for about one year; he then returned to this city, where he practised for the remaining portion of his life. For the last five years he was physician to the International Railway Company. He married Edith L. Todd, of Rochester, N. Y., who survives him, together with a son of seven years.

Those of us who recall Dr. Lynde as a student at the university remember him for his singular earnestness and diligence. It can be safely said that no one in his class worked harder or seemed more fully to realise the importance of his life work. The responsibilities of his profession seemed to impress him more and more as he advanced in years. He was the youngest of his class, having graduated before he was twenty-one. Even after this he continued to add to his professional knowledge by submitting himself to hospital training. When he finally located, he decided to remove himself from former associations, such as were connected with his old social life, and took up his abode among those whose only claim upon him was the need of his professional services, and among those he built up a large practice in which he continued until his health failed him and he finally gave his time to office work. In this he persisted to the end, although much hampered by his continual suffering.

Dr. Lynde quietly and without ostentation lived up to the high ideals of professional life; he did not attend medical meetings regularly, however, on account of his health, but he nevertheless showed a truly professional spirit, was well posted and kept up with all the rapid unfoldings of medical science. He hated sham and honored truth wherever he found it. He treated, with the utmost consideration and courtesy, all who were worthy, without regard to external circumstances. The impress of his life is stamped to remain upon his associates, and, because of his genuine merit and his professional integrity and success under difficulties, there goes out from our hearts today a tribute to his memory, the best and truest we have power to offer; and to his devoted wife, and in behalf of his fatherless child we extend our heartfelt sympathy, quickened and intensified by our own sense of loss.

Dr. Lynde was a member of the Westminster Presbyterian Church and was a man to whom external things were a reaiity. His mental condition may, perhaps, be best revealed by the following poem which after his death, was found by a relative in one of his pockets:

A PRAYER.

"Let me sleep O God, in the arms of Thy faith tonight,
And let me dream in dreams of Thee!
Let me pursue my path in Thy gentle guidance,
And let my sins forgotten be!

If I lie down and not again awake—
Or if I slumbering sink to holy rest,
Take me as I am; forgive, O God, my mortal unbelief,
And make me what is best.

In Thee have I been comforted; what e'er has been my wrong,
Whatever shall have been my mortal sin,
Most gracious Lord be not unkind to me,
But when I knock let me be welcomed in!"

Dr. Edward Clark read the following tribute to

A. T. O'Hara, M. D.

Arthur T. O'Hara was born in the city of Chicago, Ill., October 1, 1861. He attended school at the Lake Forest Academy in that city until 14 years of age, when he entered a commercial and business college and took a thorough course in bookkeeping, graduating therefrom when about 17 years of age. He at once secured employment as a bookkeeper at the stock yards in Chicago, where he remained for a period of about five years. At the end of this time he secured employment with the Panama Canal Company and spent two years in Panama, serving in the capacity of paymaster, when he contracted Panama fever; when convalescent he was sent by ship to New York. He was ill for some weeks in New York, and while there under medical care he first conceived the idea that he would like to study medicine himself. He went to Albion, N. Y., and spent one year studying in the office of Dr. H. W. Lewis. He then came to Buffalo and continued his medical studies, graduating from the medical department of the University of Buffalo in 1889. After his graduation he went to Syracuse, N. Y., where he practised his profession for one year. He then returned to Buffalo to take the civil service examination for keeper of the Quarantine Hospital. He passed the examination and secured the appointment and held the position up to the time of his death.

Dr. O'Hara was an interesting personality, and beneath his rough and grotesque exterior was found a heart overflowing with kindness, good nature and sympathy. Most of his life work in his profession consisted in ministering to the wants and necessities of the unfortunate victims of smallpox, who were sent to the Quarantine Hospital, and how well and thoroughly that work was done is evidenced by the fact that his patients all loved him for his self-sacrificing labors in their behalf.

The position which Dr. O'Hara held of necessity prevented his mingling much in social and literary affairs, so that he did not become well known in the profession, but to those of us whose duties compelled us to see him often and to know him well, he was appreciated as a bright, conscientious, intelligent and well-read physician. In his death which occurred, from rheumatism affecting the heart, on January 1, 1905, the medical profession and this society have lost an intelligent, worthy member, one whose heart was overflowing with the milk of human kindness, and one who has left the world better for his having lived in it.

